# Prevalence of tonsillar human papillomavirus infection in low-risk individuals without cancer

**DOI:** 10.31744/einstein_journal/2025AO1715

**Published:** 2025-06-03

**Authors:** Jéssica Boscariol da Silva, Aline Oliveira Silva, Luciana Reis Rosa Sacoman, Leandro Luongo Matos, Ronaldo Frizzarini, Raquel Ajub Moyses, José Eduardo Levi

**Affiliations:** 1 Tropical Medicine Institute Faculdade de Medicina Universidade de São Paulo São Paulo SP Brazil Tropical Medicine Institute, Faculdade de Medicina, Universidade de São Paulo, São Paulo, SP, Brazil.; 2 Head and Neck Surgery Department Faculdade de Medicina Universidade de São Paulo São Paulo SP Brazil Head and Neck Surgery Department, Laboratório de Investigação Médica 28 (LIM28), Faculdade de Medicina, Universidade de São Paulo, São Paulo, SP, Brazil.; 3 Faculdade de Medicina Universidade de São Paulo São Paulo SP Brazil Otorhinolaryngology Department, Faculdade de Medicina, Universidade de São Paulo, São Paulo, SP, Brazil.; 4 Department of Molecular Biology Dasa Laboratório Barueri SP Brazil Department of Molecular Biology, Dasa Laboratório, Barueri, SP, Brazil.

**Keywords:** Human papillomavirus viruses, Oropharynx, Palatine tonsil, Prevalence, Population characteristics, Surveys and questionnaires

## Abstract

**Objective:**

This study aimed to determine the prevalence of human papillomavirus (HPV) 16 infection in the tonsils of patients without cancer and to analyze the associated risk factors in a low-risk population in Brazil.

**Methods:**

The study included 113 adult patients who underwent surgery for non-neoplastic conditions. The participants completed an interview-based questionnaire on lifestyle habits, including the use of tobacco and alcohol, and a questionnaire on sexual behavior. Overall, 226 freshly frozen tonsil samples were subjected to DNA extraction, conventional polymerase chain reaction using the PGMY09/11 primer for β-globin, and quantitative polymerase chain reaction for HPV16 detection.

**Results:**

In this cohort, 47 patients (41.6%) were women between 18 and 58 years of age (mean age, 28.5 years), and 66 (58.4%) were men between 18 and 68 years of age (mean age, 34 years). A total of 81 (71.7%) and 46 (40.7%) patients answered questionnaires on lifestyle habits and sexual behavior, respectively. All samples successfully amplified the β-globin gene fragment but tested negative for HPV DNA. This outcome precluded an investigation of the association between risk factors such as sexual behavior and HPV infection. External validation using oropharyngeal squamous cell carcinoma specimens confirmed the reliability of the laboratory procedures to detect HPV DNA, showing a 10% prevalence of HPV infection in the comparator population.

**Conclusion:**

The findings of this study suggest that the actual prevalence of HPV16 tonsillar infection in the general population without cancer may be lower than that previously reported in Brazil.

## In Brief

This study investigated the prevalence of HPV16 infection in the tonsils of healthy Brazilian adults without cancer. The study included 113 patients who underwent non-neoplastic surgery. Despite using highly sensitive PCR and qPCR, HPV16 DNA was not detected in any of the 226 tonsil specimens. External validation using 38 oropharyngeal squamous cell carcinoma specimens demonstrated a 10% prevalence of HPV16 infection in this comparative population. These results suggest that the actual prevalence of HPV16 tonsillar infection in the Brazilian population may be lower than that previously reported.This study highlights the geographic variability in HPV prevalence and underscores the importance of sample collection methods and population characteristics in determining infection rates.

## Highlights

■ Prevalence of HPV16 is extremely low in the tonsil of healthy Brazilian population.

■ The prevalence of HPV16 in oropharyngeal squamous cell carcinoma was 10% in Brazilian samples.

■ The rate of HPV16 tonsillar infection should be lower than that reported previously in Brazil.

## Graphical abstract

**Figure:**
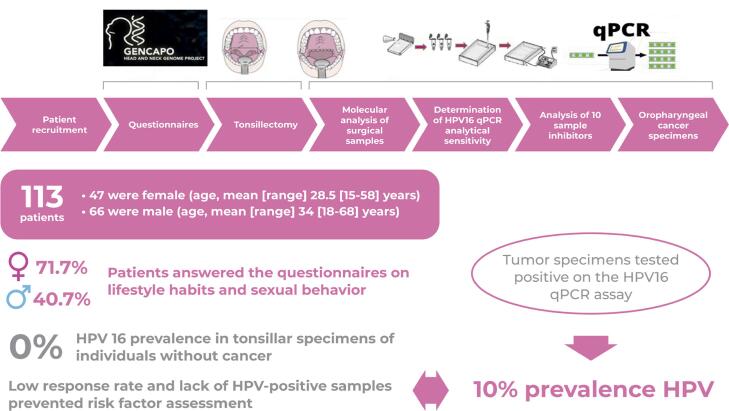

## INTRODUCTION

Human papillomavirus (HPV) infection, which has a high prevalence in both sexes, is the most common sexually transmitted infection worldwide. Approximately 75-80% of the sexually active population is infected by one or more HPV genotypes during their lifetime. Oral HPV infection is associated with an estimated 72% of the oropharyngeal squamous cell carcinomas (OPSCC) in North America.^(1)^ The incidence of chronic HPV infection-induced OPSCC has increased rapidly in the United States and possibly exceeds the incidence of HPV-induced cervical cancer.^(1,2)^ In contrast, South America has lower rates of HPV-associated cancers.^(3)^

Few studies have examined the detection of HPV DNA in normal tissues of the head and neck region obtained from healthy participants. Biopsy or tonsillectomy specimens can improve the polymerase chain reaction (PCR)-based detection of HPV DNA from the tonsils.^(4,5)^ Although it remains unclear whether HPV infection increases the risk of squamous cell carcinoma of the head and neck in healthy individuals, the tonsils appear to serve as HPV reservoirs.^(4,6)^ HPV tropism to the tonsils, where it inhabits the biofilm in the crypts and the peritonsillar extracellular space, can be explained by the ease of viral access to the invaginated crypt epithelium and by cytokine production from the lymphoid tissue, which stimulates viral transcription and cellular transformation.^(4,6)^ Further investigations into the etiopathogenesis of HPV infection are necessary because the mechanism underlying HPV-induced neoplasia, particularly in the tonsils, remains unknown, and no screening test currently exists to facilitate the early detection of HPV infection and prevention of increasingly prevalent HPV-induced cancers.^(6)^ Studying the presence of latent HPV infections in the tonsillar tissue of healthy adults can improve our understanding of HPV-associated oropharyngeal carcinomas.

## OBJECTIVE

This study aimed to determine the prevalence of HPV 16 infection in the tonsils of individuals without cancer in a low-risk population and to identify the risk factors associated with tonsillar HPV infection.

## METHODS

### Study design and participants

This prospective multicenter study included healthy adults (age ≥18 years) scheduled to undergo uvulopalatopharyngoplasty or tonsillectomy at the Otorhinolaryngology Department of the *Hospital das Clínicas, Faculdade de Medicina da Universidade de São Paulo* (HCFMUSP, a tertiary-care public university hospital and referral center), and *Hospital Paulista de São Paulo* (a private non-referral healthcare center). Participants had no history of cancer or high risk of oral HPV infection. The risk of HPV infection was indirectly inferred based on two factors: first, the geographic region where the research was conducted had a low prevalence of oral HPV infection, and second, there were no “comorbidities,” such as HIV co-infection, which may have increased this prevalence. Patients with a history of cutaneous or mucosal HPV-related lesions or mucosal cancer were excluded from the study.

Tonsillectomy is indicated primarily for obstructive sleep-disordered breathing caused by tonsil hypertrophy, which leads to snoring or disrupted sleep and frequently warrants surgical intervention. Recurrent tonsillitis, which is characterized by frequent infections that interfere with daily activities or work, and chronic tonsillitis, presenting as a persistent sore throat, halitosis, and enlarged tonsils unresponsive to medical therapy, were also key indications. Furthermore, patients who experienced symptomatic tonsillolithiasis, such as recurrent pain, irritation, or halitosis owing to tonsil stones, were considered suitable candidates for the procedure to improve their quality of life.

All participants completed two questionnaires specifically developed by a team of experienced epidemiologists as part of an international consortium (GENCAPO Study Group - *Genoma do Câncer de Cabeça e Pescoço*) investigating risk factors for head and neck cancer and also HPV-related infections. The questionnaire was designed to collect comprehensive data in the following areas: demographic characteristics, clinical history, lifestyle habits (tobacco use, alcohol consumption, hot beverage intake, and dietary habits), oral health, family history of cancer, and sexual behavior history. These domains were selected to profile the study population, compare participants from two institutions (one public and one private), and explore potential factors associated with HPV-16 exposure in the tonsils of high-risk young adults. Additionally, in cases where HPV-16 infection was detected, these data were analyzed to explore potential associations between lifestyle factors, clinical history, and HPV-16 exposure.

Trained researchers conducted face-to-face interviews to administer the questionnaire, ensure consistency in data collection, and minimize the misinterpretation of questions. Responses were recorded in a standardized format for subsequent analyses. Participants were informed that they could skip any questions they felt uncomfortable answering, particularly those about sensitive topics such as sexual behavior, respect for their privacy, and reduce potential embarrassment. Interviews were conducted privately with an assurance of confidentiality, and non-responses did not affect participation or access to care. This approach prioritizes the participants’ well-being while ensuring the collection of accurate and reliable data in other areas.

### Collection of normal oropharyngeal samples

Tonsillectomy specimens were vertically transected to obtain duplicate samples of each tonsil (right and left; four specimens per participant). Each sample was placed in a sterile 2mL cryotube, transported in liquid nitrogen to the laboratory, and stored at −80°C freezer until further testing.

### Ethics statement

This study was approved by the Ethics and Research Committee of the *Hospital das Clínicas, Faculdade de Medicina, Universidade de São Paulo* (CAAE: 01353812.0.0000.0068; #173.181). All participants provided written informed consent before enrolment. Oropharyngeal cancer samples from another study conducted at the same university hospital were also approved by the local ethics committee (CAAE: 25884914.8.0000.0068; #569.792) and evaluated in this study.

### Laboratory-based study procedures

#### DNA extraction

DNA was extracted from tonsillar specimens using the Qiagen DNeasy Blood & Tissue Kit (Qiagen, São Paulo, Brazil) according to the manufacturer’s instructions. The extracted DNA samples were quantified on the NanoDrop 2000/2000C spectrophotometer (Thermo Scientific, Wilmington, Delaware, USA). The concentration and purity were checked, and the samples were diluted to 50ng/µL and stored at −20°C.

#### HPV detection by conventional PCR

To determine the integrity of the DNA extracted from normal tissue specimens and to verify the absence of PCR inhibitors, an input sample of 250 ng DNA was subjected to amplification of a 268-bp fragment from the β-globin gene that was limited by the primers PCO4 and GH20.^(7)^ PCR, which is considered the gold standard for mucosal HPV detection,^(7)^was performed on the same sample using the primers PGMY09/11 to amplify a 450-bp fragment from the L1 region of different HPV genotypes.

#### HPV16 quantitative polymerase chain reaction (qPCR)

For all tonsillectomy samples, HPV16-specific qPCR was performed using the TaqMan Universal PCR Master Mix 2x (Applied Biosystems, Inc., USA), with a primer pair described by Walboomers et al.^(8)^ and another probe developed in our laboratory, specific to the HPV16 E7 region.^(9)^ The qPCR was performed on the QuantStudio^®^5 Real-Time PCR System (ThermoFisher Scientific, Waltham, Massachusetts, EUA) thermocycler.

DNA extracted from SiHa cells (1-2 copies of HPV16 per cell) and CaSki cells (600 copies of HPV16 per cell) was used as a positive control, while water was used as a negative control.

#### Determination of HPV16 qPCR analytical sensitivity

To simulate a putative HPV16+ tonsil, serial dilutions of CasKi cell DNA were performed in a background of HPV-negative human tonsillar DNA (50µg/µL). Ten replicates of each dilution point, corresponding to 3-3 × 10^7^ HPV 16 copies/cell, were subjected to the qPCR assay, and the proportions of positive and negative replicates were recorded and converted into a 95% hit rate using Probit analysis.

## Analysis of sample inhibitors

To rule out the presence of potential PCR inhibitors in the sample, conventional PCR and HPV16 qPCR were performed on 10 HPV-negative DNA study samples spiked with DNA from CaSki cells at a final concentration corresponding to 1.5 copies of HPV16 per cell. The spiked samples were analyzed in duplicate using HPV16 qPCR.

## Oropharyngeal cancer specimens

To confirm the effectiveness of the methodology used in this study for detecting HPV in oropharyngeal tissues, the same conditions and protocols were applied to 38 fresh-frozen OPSCC specimens with unknown HPV status, as used for the “normal” tonsillar specimens.

## Statistical analysis

Based on a published Brazilian study^(6)^ analyzing oropharyngeal swab samples from healthy individuals, we calculated that 214 duplicate samples from 107 healthy individuals were required to ensure 95% accuracy while estimating an HPV prevalence of 7.5%. All statistical analyses were conducted using the Wilcoxon rank-sum test, Student’s *t*-test, Pearson’s chi-square test, or Fisher’s exact test. Linear regression analysis was used for comparisons.

## RESULTS

In this study cohort of 113 patients, 47 were women (mean age [range] 28.5 [18-58] years), and 66 were men (mean age [range] 34 [18-68] years). Overall, 81 (71.7%) and 46 (40.7%) patients answered questionnaires on lifestyle habits and sexual behavior, respectively. Data on race, education, smoking, alcohol consumption, and sexual behavior of the participants are shown in table 1. Overall, 226 samples underwent molecular testing, including one right and one left fragment of each patient. All samples tested positive for the β-globin constitutive gene and negative for HPV DNA using generic primers PGMY09/11 and the HPV16-specific qPCR assay. Probit analysis showed an analytical sensitivity of 0.0002 copies per cell or one HPV16 copy/5,000 cells was achieved (95% hit rate). Linear regression analysis yielded a correlation index of 0.991 for the HPV16 qPCR assay. No PCR inhibitors were observed, as all 10 samples subjected to duplicate HPV16 qPCR assays tested positive.

**Table 1:** Characteristics of the study population

Variables	Results*
Public hospital, n (%)	48 (42.5)
Private hospital, n (%)	65 (57.5)
Sex, n (%)	
Women	47 (41.6)
Men	66 (58.4)
Mean age (years)	
Women	28.5 (18–58)
Men	34 (18–68)
Response to life habits questionnaire, n (%)	
Yes	81 (71.7)
No	32 (28.3)
Response to the sexual behavior questionnaire	
Yes	46 (40.7)
No	67 (59.3)
Ethnicity, n (%)	
Asian	2 (1.8)
White	66 (58.4)
Black	11 (9.8)
Mulatto	16 (14.5)
Education level, n (%)	
No education	1 (0.9)
Incomplete elementary school	9 (7.9)
Elementary school	13 (11.5)
Full high school complete	50 (44.2)
Higher education	24 (21.2)
Tobacco smoking, n (%)	
Yes	4 (3.5)
No	65 (57.5)
Only in the past	12 (10.7)
Alcohol consumption, n (%)	
Yes	37 (32.8)
No	37 (32.8)
Only in the past	5 (4.4)
Ever had sex (vaginal, oral, or anal), n (%)	
Yes	43 (93.5)
No	3 (6.5)
Oral sex (active), n (%)	
Yes	34 (73.9)
No	11 (23.9)

* Only valid cases were considered.

In the oropharyngeal cancer cohort, all 38 oropharyngeal tumor specimens tested positive for β-globin. Four of the oropharyngeal tumor specimens tested positive on PCR with the primer PGMY09/11, and two of these specimens tested positive on the HPV16 qPCR assay, corresponding to a 10% HPV prevalence.

Data on sex, age, sexual behavior, smoking, and alcohol consumption stratified according to the recruiting center are listed in table 2. Lifestyle habits differed among patients from different participating hospitals. Patients enrolled from the private hospital received education for a longer duration (years) than those recruited from the public hospital. Furthermore, participants from public hospital had a higher number of total and regular sexual partners (median, n=10 and n=4, respectively) than those from private hospital (median, n=3 and n=2 respectively). Moreover, patients enrolled in public hospital reported higher alcohol consumption.

**Table 2:** Demographic and habits data according to recruitment site

Variables	Public hospital	Private hospital	p value
Sex (n=113), n (%)			0.0332^†^
Women	14 (29.2)	33 (50.8)	
Men	34 (70.8)	32 (49.2)	
Age (median/IIQ) (years; n=113)			0.0001^£^
Median (IQR) (25–75%)	36 (29–46.7)	26 (20–33)	
Minimum – maximum	19 – 62	18 – 57	
Education (n=97; 85.8%), n (%)			0.00025^†^
No education	1 (2.1)	0 (0)	
Incomplete elementary school	8 (16.7)	1 (2.0)	
Elementary school	11 (22.9)	2 (4.1)	
High school complete	22 (45.8)	28 (57.1)	
Higher education	6 (12.5)	18 (36.7)	
Smoking history pack-years (n=29; 25.7%)			0.1825^£^
Median (IQR) (25–75%)	1.6 (0–14,35)	0 (0–2.4)	
Minimum – maximum	0 – 20.5	0 – 12.7	
Alcoholism history (grams per day; n=80; 70.8%)			0.0042^£^
Median (IQR: p25–p75%)	53.3 (1.5–661)	0 (0–81.9)	
Minimum – maximum	0–34,973.3	0–49,90.9	
Sexual behavior, n (%)			
Response to the sexual habit questionnaire (n=113)			0.5679^†^
Yes	18 (37.5)	28 (43.1)	
No	30 (62.5)	37 (56.9)	
Genital’s warts (n=46; 40.7%)			1.0^†^
I do not know	0 (0)	1 (3.6)	
No	18 (100)	27 (96.4)	
**Variables**	**Public hospital**	**Private hospital**	**p value**
Genital HPV (n=46; 40.7%)			0.7200^‡^
Yes	2 (11.1)	3 (10.7)	
I do not know	0 (0)	1 (3.6)	
No	16 (88.9)	24 (85.7)	
Ever had sex (vaginal, oral, or anal) (n=46; 40.7%), n (%)			0.2696^†^
Yes	18 (100)	25 (89.3)	
No	0 (0)	3 (10.7)	
Number of lifetime sex partners – any sex (n=35; 31%)			0.0018^£^
Median (IQR: p25–p75%)	10 (5–19)	3 (2–6)	
Minimum – maximum	1 – 50	0 – 10	
Number of lifetime sex partners – any sex (n=46; 40.7%), n (%)			0.2973^‡^
0	0 (0)	3 (10.7)	
1	1 (5.6)	2 (7.1)	
2–5	5 (27.8)	12 (42.9)	
6–10	6 (33.3)	7 (25)	
11–20	4 (22.2)	1 (3.6)	
21–50	2 (11.1)	2 (7.1)	
51–100	0 (0)	1 (3.6)	
Number of regular sex partners (n=38; 33.6%)			0.1978^£^
Median (IQR: p25–p75%)	3 (1–8)	2 (1–5)	
Minimum – maximum	1 – 12	1 – 8	
Number of casual sex partners (n=36; 31.9%)	14	22	0.0175^£^
Median (IQR: p25–p75%)	4 (1.7–10,5)	1.5 (0–4)	
Minimum – maximum	0 – 38	0 – 7	
How many times have you paid or received money or drugs to have sex (n=43; 38.1%)			0.0366^£^
Median (IQR: p25–p75%)	0 (0–0)	0 (0–0)	
Minimum – maximum	0 – 4	0 – 0	
Oral sex (active) (n=46; 40.7%)			0.1681^‡^
Yes	16 (88.9)	18 (64.3)	
No	2 (11.1)	9 (32.1)	
Not reported	0 (0)	1 (3.6)	
Age first performed oral sex (years; n=31; 27.4%)			0.2077^£^
Median (IQR: p25– p75%)	18 (17–22)	18 (16–19)	
Minimum – maximum	13 – 30	15 – 22	
Monthly frequency of oral sex (n=35; 31%)			0.6473^£^
Median (IQR: p25–p75%)	2 (2–8)	3 (1–8)	
Minimum – maximum	1 – 12	1 – 12	
Sex of the partners in whom he performed oral sex (n=35; 31%)			0.1841^‡^
Women	11 (68.8)	8 (42.1)	
Men	5 (31.3)	9 (47.4)	
Both sexes	0 (0)	2 (10.5)	
Oral sex (passive) (n=46; 40.7%), n (%)			0.2650^‡^
Yes	14 (77.8)	16 (57.1)	
No	4 (22.2)	10 (35.7)	
Not reported	0 (0)	2 (7.1)	
Mouth on partner anus (n=46; 40.7%), n (%)			1.0^†^
Yes	3 (16.7)	4 (14.3)	
No	15 (83.3)	24 (85.7)	

^†^ Fischer’s exact test; ^‡^ Pearson χ^2^ test; ^£^ Wilcoxon test.

IQR: Interquartile ranges; HPV: Human Papilloma Virus.

## DISCUSSION

The detection of asymptomatic chronic HPV16 infection, followed by the development of precursor lesions that progress to cancer, contributes to the establishment of a theoretical model of OPSCC that is analogous to that of HPV-driven cervical carcinogenesis. As OPSCC occurs more often in young individuals who may acquire HPV16 through oral sex, we evaluated freshly frozen samples obtained from the tonsils, the oropharyngeal site most commonly associated with HPV16 infection. The results of similar studies on the rate of HPV detection show wide variations, which is likely due to differences in the methods of collection, detection of viral DNA, demographics of the study population, and geographic region.^(9-13)^

We considered the possibility that the prevalence of HPV-driven oropharyngeal cancers varies geographically. In developed countries, approximately 40% of OPSCC cases, mainly tonsillar cancer, are attributed to HPV, whereas in Latin American countries, the prevalence of HPV-induced OPSCC is <20%. In Brazil, the prevalence of HPV16-associated oropharyngeal cancer is only 4%, which explains the low HPV16 prevalence in our study population.^(14,15)^ None of the healthy participants tested positive for HPV. The methodology for HPV detection was carefully reviewed, and cancer specimens were included to ascertain the accuracy of the HPV detection technique. The sample size estimation was based on a study by Sacramento et al.,^(6)^ which identified an HPV prevalence of 7.5% in a Brazilian cohort of healthy participants. A systematic review^(16)^ that included all Brazilian studies published up to 2014 identified a 6.2% prevalence of oral or oropharyngeal HPV infection in the healthy general population. However, two of the included studies (13.3%) found no HPV-positive cases among 204 eligible participants, and their results are similar to the results of this study. Based on these findings, the absence of HPV infection in the selected sample is plausible.

Thus, the overall prevalence of HPV infections in the Brazilian population may vary. According to a systematic review of Brazilian studies that included 2,060 patients without cancer, the detection rate of any HPV type varied from 0.0 to 29.2%, with a pooled positivity rate of 6.2% for oral and oropharyngeal HPV infections. When tested specifically for HPV16, 22 of the 1,551 (1.4%) participants were HPV16-positive, varying from 0 to 20.8%.^(16)^

Another relevant factor is the type of collection and samples analyzed in population studies. The Study of Natural History of Human Papillomavirus Infection and Precancerous Lesions in the Tonsils (SPLIT) was the first to evaluate HPV prevalence in participants without tonsillar neoplasia in exfoliated cells using brushings of tonsillectomy specimens and gargles of the same immunocompetent patients.^(10)^ The prevalence of HPV infection, as determined from tonsillectomy specimen brushings and gargles, was 3.6% and 13.1%, respectively. The percentage agreement and positive concordance of HPV detection in paired tonsillectomy brushings and gargles in adults were 85.8% and 9.5%, respectively, suggesting that gargles are not representative biological specimens for determining the prevalence of viral infection.^(10)^ Another study comparing brushing and biopsy specimens for oral and oropharyngeal lesions highlighted the high inadequacy of samples obtained by brushing.^(17)^ In our study, to ensure sample preservation and maintain DNA integrity, tonsillectomy specimens were collected immediately after resection in the surgical center and frozen at −80°C.

For a long time, differences in HPV detection methodologies have been discussed, as several tests with variable sensitivities have been employed.^(6,12,18)^ However, we are confident that the absence of tonsillar HPV DNA in the current investigation was not due to technical issues, such as PCR sensitivity or inhibitors. Our study-related experiments included spiking previously HPV-negative samples with a low concentration of HPV16 DNA, which was readily detected in further testing. Moreover, when OPSCC specimens were subjected to the same procedure, 10% of the samples were HPV-positive, and two contained HPV16 DNA. This provides confirmatory evidence that this study was conducted in accordance with all requirements for optimal DNA preservation and control for accurate viral DNA detection.

In addition to HPV DNA testing, this study collected data on risk behaviors in a cancer-free population to provide useful information for designing and conducting primary HPV infection prevention campaigns. Although fewer participants completed the sexual behavior questionnaire, data analysis revealed that participants from the public hospital had a higher number of total and casual sexual partners (10 and 4, respectively). However, the reasons for refusal to answer the sexual behavior questionnaire may not have been random and could have implications for the results regarding sexual behavior. Discussions about HPV and sexual behavior should be approached sensitively and, if possible, individually to prevent embarrassment and facilitate a higher response rate to the sexual behavior questionnaire.^(19)^

The evaluation of oral sexual practices by the participants was limited by the very low response rate; however, several studies have identified sexual practices as the main factor for HPV transmission among adults.^(14,20)^ We observed that patients with genital HPV infection had more sexual partners than those without infection, but the difference was not statistically significant. Gillison et al.^(21)^ evaluated the association between the number of sexual partners and genital HPV infection in the USA and reported a higher prevalence of oral HPV among participants with >20 sexual partners during their lifetime. The differences in the educational and sexual behavior profiles of the participants recruited from the two hospitals underscore the importance of adequate demographic representation in studies on HPV prevalence in the Brazilian population.

However, this study has some limitations. First, although we performed a technically correct sample calculation, the sample size for the statistical analysis may have been underestimated. This may be attributable to the possibility that the study we used as the basis for the sample size calculation^(6)^ could have evaluated a higher-risk population than ours or possibly overestimated HPV prevalence. Second, only HPV 16 was evaluated, which may have biased the prevalence of other subtypes, leading to no evidence of “HPV” in any sample. Third, although the questionnaires were not designed as standardized tools for widespread use, their content can serve as a valuable reference for future studies exploring similar research questions. Additionally, the low number of responses to the sexual behavior questionnaire, possibly due to embarrassment induced by the interviewer-based questionnaire survey, may have introduced information bias. Many studies have shown that self-administered questionnaires^(21)^ can improve the response rates to intimate questions.

## CONCLUSION

This study showed that the HPV 16 prevalence in tonsillar specimens of individuals without cancer was 0%. However, the low response rate to the sexual behavior questionnaire and the absence of HPV-positive biological samples prevented the assessment of potential risk factors associated with HPV infection.
